# ATR mediates cisplatin resistance in 3D-cultured breast cancer cells via translesion DNA synthesis modulation

**DOI:** 10.1038/s41419-019-1689-8

**Published:** 2019-06-12

**Authors:** Luciana Rodrigues Gomes, Clarissa Ribeiro Reily Rocha, Davi Jardim Martins, Ana Paula Zen Petisco Fiore, Gabriela Sarti Kinker, Alexandre Bruni-Cardoso, Carlos Frederico Martins Menck

**Affiliations:** 1 Departamento de Microbiologia, Instituto de Ciências Biomédicas, Universidade de São Paulo, São Paulo, SP Brazil; 2 Departamento de Bioquímica, Instituto de Química, Universidade de São Paulo, São Paulo, SP Brazil; 3 Departamento de Fisiologia, Instituto de Biologia, Universidade de São Paulo, São Paulo, SP Brazil; 4 Present Address: Laboratório Especial de Ciclo Celular, Instituto Butantan, São Paulo, SP Brazil; 5 Present Address: Departamento de Oncologia Clínica e Experimental, Escola Paulista de Medicina, Universidade Federal de São Paulo, São Paulo, SP Brazil

**Keywords:** Breast cancer, Cancer microenvironment, Mechanisms of disease, DNA damage checkpoints

## Abstract

Tissue architecture and cell–extracellular matrix (cell–ECM) interaction determine the organ specificity; however, the influences of these factors on anticancer drugs preclinical studies are highly neglected. For considering such aspects, three-dimensional (3D) cell culture models are relevant tools for accurate analysis of cellular responses to chemotherapy. Here we compared the MCF-7 breast cancer cells responses to cisplatin in traditional two-dimensional (2D) and in 3D-reconstituted basement membrane (3D-rBM) cell culture models. The results showed a substantial increase of cisplatin resistance mediated by 3D microenvironment. This phenotype was independent of p53 status and autophagy activity and was also observed for other cellular models, including lung cancer cells. Such strong decrease on cellular sensitivity was not due to differences on drug-induced DNA damage, since similar levels of γ-H2AX and cisplatin–DNA adducts were detected under both conditions. However, the processing of these cisplatin-induced DNA lesions was very different in 2D and 3D cultures. Unlike cells in monolayer, cisplatin-induced DNA damage is persistent in 3D-cultured cells, which, consequently, led to high senescence induction. Moreover, only 3D-cultured cells were able to progress through S cell cycle phase, with unaffected replication fork progression, due to the upregulation of translesion (TLS) DNA polymerase expression and activation of the ATR-Chk1 pathway. Co-treatment with VE-821, a pharmacological inhibitor of ATR, blocked the 3D-mediated changes on cisplatin response, including low sensitivity and high TLS capacity. In addition, ATR inhibition also reverted induction of REV3L by cisplatin treatment. By using REV3L-deficient cells, we showed that this TLS DNA polymerase is essential for the cisplatin sensitization effect mediated by VE-821. Altogether, our results demonstrate that 3D-cell architecture-associated resistance to cisplatin is due to an efficient induction of REV3L and TLS, dependent of ATR. Thus co-treatment with ATR inhibitors might be a promising strategy for enhancement of cisplatin treatment efficiency in breast cancer patients.

## Introduction

Breast cancer is a major public health problem due to its high incidence and mortality^1^. The search for new drugs is still an active field on oncology research; however, it is common that results for drug efficiency obtained in vitro are not reproduced in the clinic. A possible explanation for this hindered efficiency is the fact that most preclinical studies are conducted in monolayer cell culture models, in which tumor cell lines are exposed to the drug to be tested in a two-dimensional (2D) plastic surface, completely ignoring the surrounding microenvironment influences.

In vivo, cells are embedded in a complex three-dimensional (3D) microenvironment, whose cellular and chemical composition is tissue specific. The cell–extracellular matrix (ECM) contact establishes cellular polarity and tissue architecture. Consequently, it defines the organ specificity and function^2^. In pathological conditions, such as cancer, cellular microenvironment also displays a central role. Thus tumorigenesis and cancer progression are not tumor cell-autonomous processes, but they can be influenced, or even driven, by the crosstalk between tumor cells and its surrounding microenvironment^3^. Consequently, cell–microenvironment interaction also exerts a crucial impact on controlling anticancer drugs response^4^. Although the traditional monolayer cell culture model has allowed significant advances in cancer understanding, this methodology has many limitations and might not faithfully capture the actual tumor behavior^5^. In turn, by mimicking fundamental aspects of the in vivo microenvironment, 3D cell culture models might provide more assertive conclusion regarding the effectiveness of chemotherapy and emerge as more robust methodologies for drug screening analysis^5^.

Here we examined the 3D microenvironment effect on breast cancer cell response to cisplatin. This anticancer drug is one of the most effective antitumor agents for treatment of several tumor types (ovarian, testicular, lung, and others), but not for breast cancer^6–8^. Divergence on the mutation profile of cancer cells is not enough to explain why the same drug induces such different responses. Despite its remarkable efficiency, most tumors display intrinsic or acquired resistance to chemotherapy^9^. Several molecular mechanisms of tumor cell resistance to cisplatin have been described, such as increased DNA damage response (DDR) signaling and improved translesion DNA synthesis (TLS)^9–13^.

We demonstrated here that human breast cancer cells acquire substantial resistance to cisplatin when the cell–ECM contact is re-established in vitro in a 3D context. Thus, in 2D cell culture model, high doses of cisplatin induce massive cell death. On the other hand, in 3D microenvironment, cisplatin results in DNA damage-induced cellular senescence. This phenotype correlated with increased ATR-Chk1 signaling and improvement of cellular capacity of replicating cisplatin-damaged DNA. Finally, we also demonstrated that inhibition of ATR reverted several of the 3D microenvironment-mediated responses to cisplatin, increasing sensitivity of breast cancer cells to this classical anticancer drug.

## Results

### 3D-cultured breast cancer cells are much more resistant to cisplatin than 2D-cultured ones, although subject to similar levels of DNA damage

**Fig. 1:**
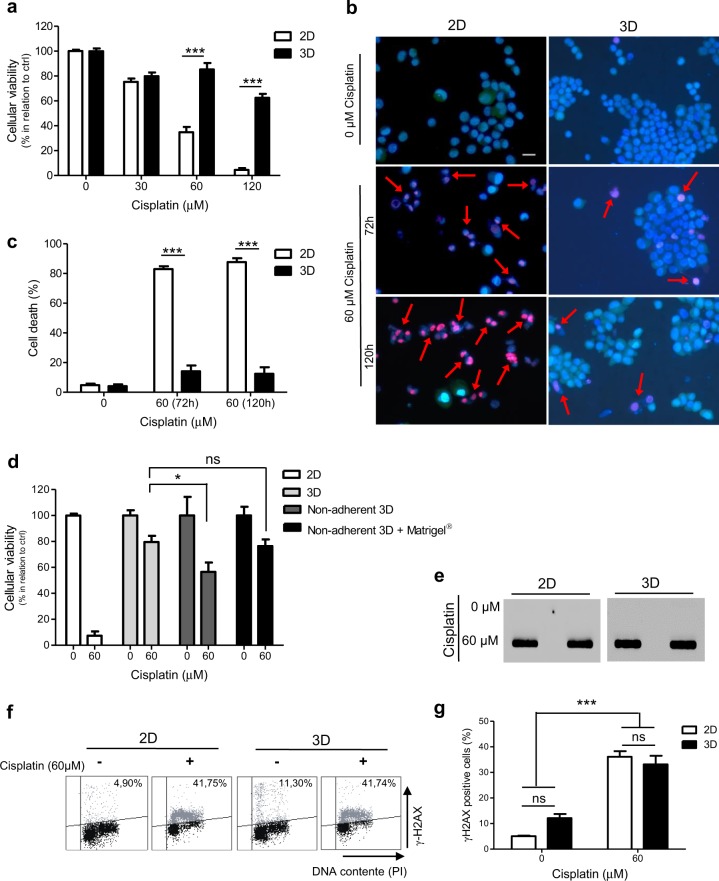
Three-dimensional (3D)-cultured MCF-7 cells are more resistant to cisplatin than two-dimensional (2D) ones, despite similar amount of induced DNA damage. **a** Cellular viability analysis of cells exposure to different doses of cisplatin for 72 h, by XTT assays. **b**, **c** Cell death assessment in cells treated with cisplatin for 72 and 120 h, by fluorescence microscopy. A combination of dyes (fluorescein di-acetate, propidium iodide, and Hoechst) was used to distinguish viable cells from those in necrosis or apoptosis. **c** Cells were counted, and the percentages were plotted on a graph. **d** Cellular viability assay, in response to 72 h of cisplatin treatment, in MCF-7 cells cultured under 2D, 3D, or non-adherent 3D (with or without media supplemented with 4% Matrigel^®^) conditions. **e** Immunodetection of cisplatin–DNA adducts, by slot-blot assays, upon 1 h of treatment. **f**, **g** γ-H2AX detection, followed by flow cytometry, for evaluation of DNA damage induced by cisplatin (4 h). In all graphs, the results are presented as mean ± SEM from at least two independent experiments performed in duplicate. Two-way analysis of variance and Bonferroni post hoc test were used for statistical analysis and the differences were considered significant for *P ≤ 0.05 and ***P ≤ 0.001. ns highlights non-significant changes. **b** The red arrows indicate necrotic cells (type of cell death mostly detected in MCF-7 cells) whose standard staining is characterized by the red nuclei and cytoplasm homogeneously stained with propidium iodide. The white scale bar correspond to 10 μM

To assess changes on cellular sensitivity to cisplatin potentially mediated by cell architecture and cell–ECM interaction, we evaluated the behavior of cisplatin-treated cells under distinct cell culture conditions: the standard monolayer culture on plastic (2D culture) versus in 3D-reconstituted basement membrane gels (3D-rBM), in which cells form micro-tissue-like structures^14^. First, we assessed the cellular viability of 2D- and 3D-cultured breast cancer MCF-7 cells upon treatment with cisplatin (Fig. 1a). We observed a drug resistance effect in cells treated under 3D context. Higher levels of cell death were also detected in cells exposed to cisplatin in 2D culture than those in 3D (Fig. 1b, c). This remarkable increase in cisplatin resistance observed in 3D-rBM cultures could be as much a result of the cell–ECM contact as due to morphological changes (formation of spherical structures) and increased cell-to-cell contact induced by growth in 3D. To address the influence of 3D architecture separate from the ECM on cisplatin responses, cells were cultured in non-adherent plates, in medium supplemented or not with rBM (Fig. 1d). Cellular viability analyses also indicate resistant phenotypes in these conditions and suggest that three-dimensionality is the most important factor influencing cisplatin resistance. However, morphological changes alone were not enough to achieve the same cisplatin resistance levels observed in 3D-rBM cultures. Only the combination of both factors, 3D-architecture and basement membrane, rescued completely the resistance to cisplatin seen in 3D-rBM cultures (Fig. 1d).

These flagrant changes on cellular sensitivity to cisplatin could be associated with potential differences on the levels of drug-induced DNA damage, in distinct cellular contexts. However, immunodetection of cisplatin–DNA adducts and γ-H2AX revealed that cisplatin induces similar levels of DNA damage in both 2D and 3D-rBM culture conditions (Fig. 1e, f).

### The differential response to cisplatin in 3D-rBM is not related to p53 status and autophagy activity

**Fig. 2:**
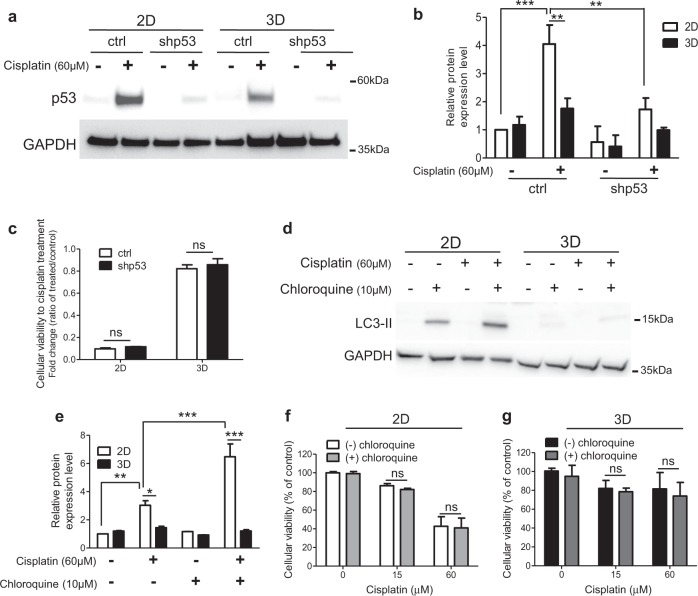
Cisplatin resistance mediated by cell growth in three-dimensional (3D) culture is independent of p53 and autophagy activity. **a**, **b** Immunoblot for p53 and GAPDH in total lysates from MCF-7 cells deficient for p53 (shp53) and shRNA scramble control (ctrl) treated with cisplatin for 24 h, under 2D and 3D culture conditions. **c** Cellular viability in response to cisplatin (60 μM for 72 h) of shp53 and ctrl MCF-7 cells under both culture conditions, by XTT assays. **d**–**g** MCF-7 cells treated with chloroquine (autophagy pharmacological inhibitor) and cisplatin, for 72 h, were used for **d**, **e** immunodetection of LC3-II and GAPDH and evaluation of cellular viability, by XTT assays, under 2D and 3D culture contexts (**f**, **g**). Densitometric analysis of p53 (**a**, **b**) and LC3-II (**d**, **e**) protein levels were normalized to the endogenous control (GAPDH). In all graphs, the results are presented as mean ± SEM from at least two independent experiments. Two-way analysis of variance and Bonferroni post hoc test were used for statistical analysis and the differences were considered significant for *P ≤ 0.05, **P ≤ 0.01 and ***P ≤ 0.001. ns highlights non-significant changes

Previous data, regarding the 3D culture effect on cellular sensitivity to doxorubicin, established the p53-dependent induction of autophagy as the central pathway responsible for the basement membrane control of chemotherapy response^4^. Here we show an increase on p53 protein expression levels in MCF-7 cells’ exposure to cisplatin under both culture conditions (Fig. 2a, b). However, this cisplatin-resulting induction of p53 was higher in 2D than in 3D culture context. To evaluate whether p53 could mediate the 3D culture-related changes on cellular susceptibility to cisplatin treatment, cells silenced for p53 expression were used (Fig. 2a, b). Non-significant variations on cellular viability of p53-deficient MCF-7 cells (shp53) were detected (Fig. 2c). Furthermore, cellular viability analyses show a similar profile of enhanced resistance to cisplatin in 3D-cultured cells and also in the p53-mutated breast cancer cells MDA-MB-231 (Fig. S1a). This p53-independent behavior was also shown in lung cancer cells (Fig. S1b, c).

Further, we considered the autophagy participation in this mechanism. We inspected potential differences on LC3-II expression level accumulation, a well-established autophagy marker, in 2D- and 3D-cultured MCF-7 cells (Fig. 2d, e). For measurement of the autophagy flux, co-treatment with chloroquine (inhibitor of autophagy) was performed. No LC3-II accumulation was observed in MCF-7 cells maintained in 3D culture context, even when co-treated with chloroquine and cisplatin. However, a significant LC3-II expression level accumulation was detected in 2D-cultured cells only on exposure to chloroquine or double treated with chloroquine and cisplatin (Fig. 2d, e). The effect of autophagy blockage on cisplatin sensitivity was also evaluated (Fig. 2f, g). Non-significant changes on cellular viability in response to cisplatin were induced by chloroquine co-treatment in both cell culture conditions.

### Cisplatin induces persistent DNA damage and senescence in 3D-rBM-cultured breast cancer cells

**Fig. 3:**
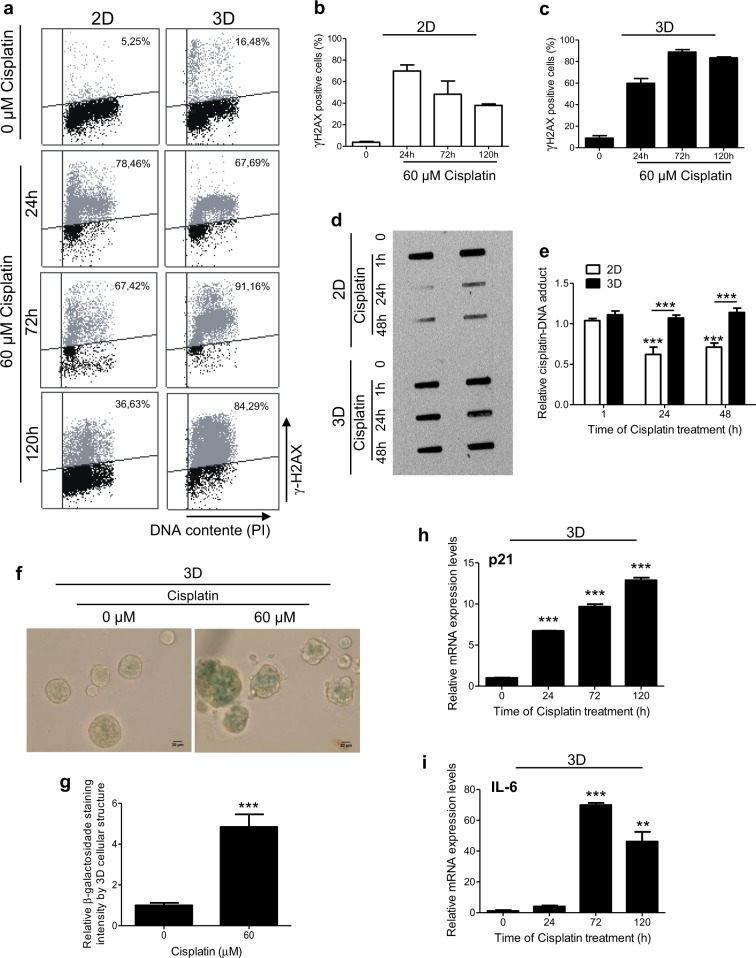
Cisplatin-induced DNA damage is not removed along of time in three-dimensional (3D)-cultured MCF-7 cells and correlates with senescence induction. **a**–**c** γ-H2AX immunodetection followed by flow cytometry and **d**, **e** cisplatin–DNA adduct detection by slot-blot assays, in cells exposure to 60 μM of cisplatin by different periods of time, for evaluation of DNA damage processing. **f**, **g** β-Galactosidase staining at pH 6 in 3D-cultured cells treated with cisplatin for 120 h. Quantitative PCR (qPCR) assays for analysis of the mRNA expression levels of (**h**) p21 and (**i**) interleukin-6 (indicators of cellular senescent phenotype) in cisplatin-treated cells under 3D cell culture condition. The expression of GAPDH mRNA was used as an endogenous control for the qPCR assays. In all graphs, the results are presented as mean ± SEM from at least two independent experiments performed in duplicate. Student *t* test (**g**), one-way analysis of variance (ANOVA) followed by Tukey post-test (**h**, **i**) and two-way ANOVA and the Bonferroni post-hoc test (**e**) were used for statistical analysis and the differences were considered significant for **P ≤ 0.01 and ***P ≤ 0.001

Further, we investigated the cisplatin-induced DNA damage processing. For this purpose, γ-H2AX levels were quantified in 2D- and 3D-cultured MCF-7 cells after exposure to cisplatin for distinct periods of time (Fig. 3a–c). While in 2D context the γ-H2AX levels decrease over time, in 3D culture conditions this standard DNA damage marker did not decline. To further evaluate DNA repair capabilities of these cells, slot-blot assays were performed for immunodetection of cisplatin–DNA adducts (Fig. 3d, e). Significant removal of cisplatin–DNA adducts was detected only in cells cultured in monolayer, while the levels of DNA damage in 3D-cultured cells did not reduce with time.

Therefore, although more resistant to cisplatin-induced cell death than 2D-cultured cells, breast cancer cells under 3D culture context present persistent DNA damage. Previous literature data have demonstrated that DNA lesion persistence can lead cells to senescence^15^. In order to confirm that cisplatin induces senescence in 3D-cultured breast cancer cells, we measured the pH-dependent activity of β-galactosidase (Fig. 3f, g). We detected significant increase of β-galactosidase staining levels at pH 6 by cisplatin treatment. β-Galactosidase staining was also detected in the few 2D-cultured MCF-7 cells that survived cisplatin treatment (data not shown). Moreover, significant increases of the mRNA expression levels of p21 (Fig. 3h) and interleukin 6 (IL-6) (Fig. 3i), important genes associated with senescence phenotype, were also detected after cisplatin exposure^16,17^.

### 3D-cultured cells treated with cisplatin progress through S cell cycle phase and do not present impairment of DNA replication

**Fig. 4:**
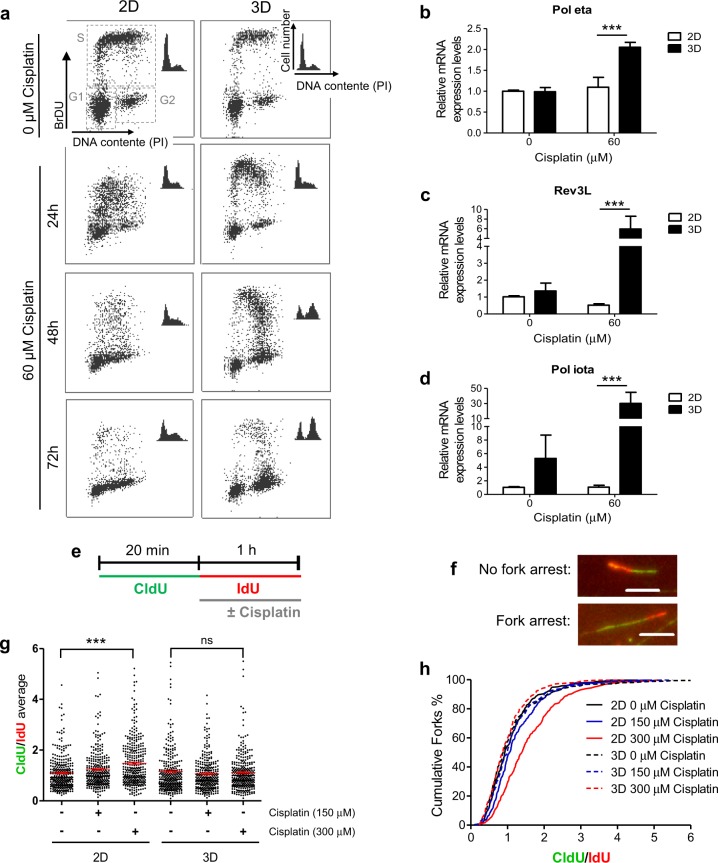
Cisplatin effects on cell cycle progression, translesion DNA polymerases expression, and replication fork stalling are distinct in two- and three-dimensional cell culture contexts. **a** Cell cycle analysis of cells treated with cisplatin for different periods of time, using double staining with bromodeoxyuridine and propidium iodide followed by flow cytometry. **b**–**d** Quantitative PCR (qPCR) assays for detection of mRNA expression levels of (**b**) Pol eta, (**c**) REV3L, and (**d**) Pol iota upon treatment with cisplatin (24 h). GAPDH mRNA expression was used as an endogenous control for qPCR assays. **e** Scheme of DNA fiber assays performed with cells’ exposure to distinct doses of cisplatin for 1 h. **f** Representative images of fibers obtained for not arrested and arrested replicative forks situations. The white scale bars correspond to 5 μM. **g** Average of chlorodeoxyuridine/iododeoxyuridine (CldU/IdU) ratios and (**h**) cumulative frequency distribution of the CldU/IdU ratio expressed as the percentage of cumulative forks. **g**, **h** For CldU/IdU ratios, ≥150 fibers were considered for each independent experiment. In all graphs, the results are presented as mean ± SEM from at least two independent experiments. **g** One-way analysis of variance (ANOVA) followed by Tukey post-test and (**b**–**d**) two-way ANOVA and the Bonferroni post hoc test were used for statistical analysis and the differences were considered significant for ***P ≤ 0.001. ns highlights non-significant changes

The effect of cisplatin on cell cycle distribution of 2D- and 3D-cultured breast cancer cells was further investigated (Fig. 4a and Fig. S2a, b). The results show that, upon cisplatin exposure, MCF-7 cells can progress through S and reach the G2–M cell cycle phase, only under 3D context (Fig. 4a and Fig. S2a). Upon 72 h of treatment, the percentage of cells in G2–M cell cycle phase was higher in 3D-rBM than cells in monolayer (Fig. 4a and Fig. S2b).

As cisplatin-induced cell death results from prolonged replication fork arrest and their consequent collapse, increased TLS capacity is a central mechanism underlying cisplatin resistance. This process of DNA damage tolerance is mediated by specific polymerases (TLS Pol) from Y- (Pol eta, Pol iota, Pol kappa, and REV1) and B-families (Pol zeta)^9^. These TLS Pols can bypass DNA damage preventing prolonged replicative stress and, consequently, decreasing cellular sensitivity. We hypothesized that 3D-cultured MCF-7 cells present an important TLS capacity, because only under this circumstance cisplatin-treated cells progress through the DNA synthesis cell cycle phase, despite displaying persistent DNA damage. Higher mRNA expression level induction was detected in cells under 3D culture conditions when compared to those in 2D for three out of five TLS Pols: Polymerase eta (Pol eta) (Fig. 4b), REV3L (catalytic subunit of Polymerase zeta) (Fig. 4c), and Polymerase iota (Pol iota) (Fig. 4d). Non-significant changes on REV1 (Fig. S2c) and Polymerase kappa (Pol kappa) (Fig. S2d) transcriptional levels were detected.

To better characterize the involvement of the 3D microenvironment on progression of replication forks, we performed DNA fiber assays (Fig. 4e, f). We detected a significant increase of chlorodeoxyuridine/iododeoxyuridine (CldU/IdU) ratios average, which indicates fork stalling, only for 2D-cultured MCF-7 cells treated with 300 μM of cisplatin (Fig. 4g). On the other hand, non-significant changes on replication fork speed were observed for cells treated under 3D culture condition (Fig. 4g). This 2D-exclusive profile of replication fork stalling in response to cisplatin is also shown in graphs of cumulative frequency distribution of the CldU/IdU ratio (expressed as the percentage of cumulative forks) (Fig. 4h).

### ATR inhibition impairs resistance to cisplatin by decreasing TLS capacity of 3D-cultured breast cancer cells

DDR comprises an ordered cascade of events responsible for controlling a set of cellular processes in order to maintain the genome integrity^18^. However, within an anticancer therapy context, DDR activation by tumor cells might contribute to their survival and compromise the patient’s treatment. The main mediators of DDR signaling are the phosphoinositide 3-kinase-related protein kinases, ATM (ataxia-telangiectasia mutated) and ATR (ATM- and Rad3-related), which in turn activate their downstream targets Chk2 and Chk1 (checkpoint kinase 2 and 1, respectively)^10^. ATM responds primarily to DNA double-strand breaks^19^. In turn, ATR is usually activated when RPA-coated ssDNA (replication protein A heterotrimer-coated single-strand DNA) nucleofilaments are formed in consequence of replication fork stalling^19^. Because cisplatin mainly induces ATR-dependent DDR, the use of ATR inhibitors has been tested as a promising strategy for increasing sensitivity to cisplatin^20,21^.

**Fig. 5:**
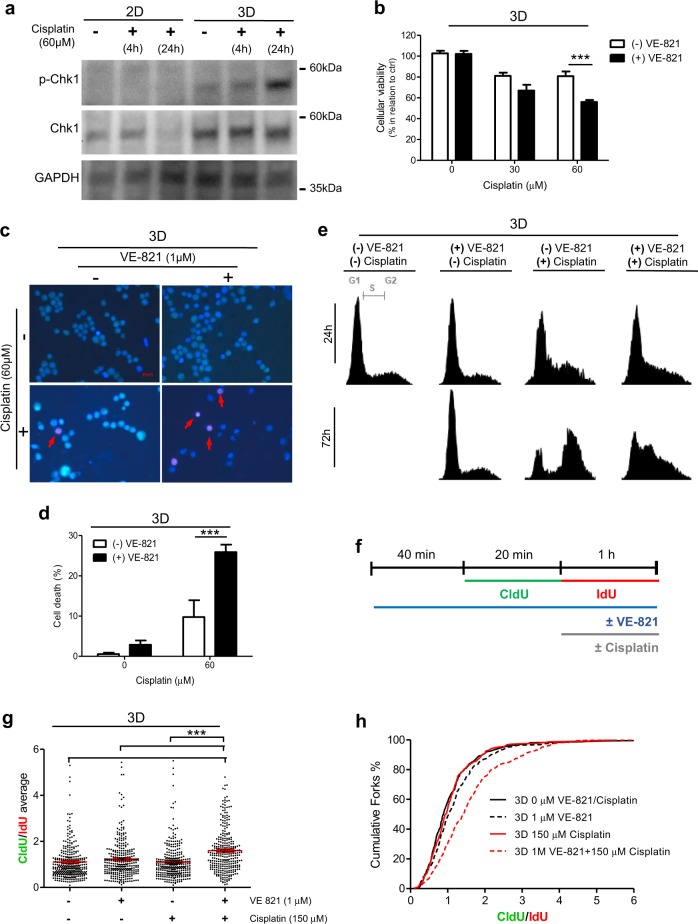
Three-dimensional (3D)-mediated effects on MCF-7 cells’ response to cisplatin are reversed by ATR inhibition. **a** Immunoblots for p-Chk1 (Ser345), total Chk1, and GAPDH (loading control) in protein lysates from cells treated with cisplatin for distinct times, under two-dimensional and 3D culture conditions. **b**–**d** 3D-cultured cells treated with 1 μM of VE-821 (ATR pharmacological inhibitor) and different doses of cisplatin for 72 h were evaluated for **b** cellular viability and cell death rates (**c**, **d**) by XTT assays and fluorescent microscopy, respectively. **e** Cell cycle analysis, through propidium iodide staining followed by flow cytometry, in 3D-cultured cells co-treated with VE-821 (1 μM) and cisplatin (60 μM) for 24 and 72 h. **f** Scheme of DNA fiber assay performed with cells treated with VE-821 (1 μM) and cisplatin (150 μM). **g** Average of chlorodeoxyuridine/iododeoxyuridine (CldU/IdU) ratios and **h** cumulative frequency distribution of the CldU/IdU ratio expressed as the percentage of cumulative forks. **g**, **h** For CldU/IdU ratios, ≥150 fibers were considered for each independent experiment. In all graphs, the results are presented as mean ± SEM from at least two independent experiments performed in duplicate. **g** One-way analysis of variance (ANOVA) followed by Tukey post-test and **b**, **d** two-way ANOVA and the Bonferroni post-hoc test were used for statistical analysis and the differences were considered significant for ***P ≤ 0.001

To investigate whether the 3D culture-mediated changes on cisplatin response are associated with distinct ATR activity, we measured the levels of Chk1 total and phosphorylated (pChk1) at serine 345 (S345), by immunoblotting, in MCF-7 cells treated with cisplatin (Fig. 5a). Densitometric analysis shows a significant induction of pChk1/Chk1 total ratio, in relation to its respective untreated control, only in cells under 3D culture context (Fig. S3a).

To determine the relevance of ATR activation on mediating the 3D-associated responses to cisplatin, we analyzed the effect of co-treatment with VE-821, a recognized ATR inhibitor^22,23^. Decrease on cellular viability was detected in 3D-cultured MCF-7 cells co-treated with VE-821 and cisplatin (Fig. 5b). Non-significant changes in cisplatin cellular viability were observed upon VE-821 addition, when MCF-7 cells were maintained in monolayer (Fig. S3b). Co-treatment with AZ20 (another ATR pharmacological inhibitor, structurally distinct from VE-821) potentiated the cisplatin effects decreasing cellular viability in both 2D and 3D culture conditions (Fig. S3c, d). The levels of cisplatin-induced cell death were also increased in 3D-rBM by ATR inhibition (Fig. 5c, d). Moreover, co-treatment with this ATR inhibitor also changes the cell cycle distribution (Fig. 5e). Distinct from 3D-cultured MCF-7 cells only treated with cisplatin, cells simultaneously exposed to VE-821 and cisplatin were not able to progress through S cell cycle phase and reach G2–M.

Further, we examined whether ATR inhibition would also be able to block the 3D-mediated effect on replication fork progression. DNA fiber assays were performed in cisplatin-treated MCF-7 cells, under 3D culture conditions, submitted to pre- and co-treatment with VE-821 (Fig. 5f). The results show that VE-821 significantly increased the average of CldU/IdU ratio obtained upon 1 h of treatment with cisplatin (Fig. 5g). Consequently, the profile of cumulative fork was altered only upon double treatment with VE-821 and cisplatin (Fig. 5h).

We evaluated whether the level of the TLS DNA Pols and the ATR pathway correlate with the 3D culture-induced resistance to cisplatin also in a breast cancer basal-like cell line (MDA-MB-231 cells). Our results suggest that the mechanism responsible for such 3D-mediated increase of cisplatin resistance is different between these cell lines. Unlike the luminal breast cancer cells (MCF-7 cells), in MDA-MB-231 cells none of the TLS DNA polymerases were induced by cisplatin treatment in either 2D or 3D cultures (Fig. S4a). In addition, the effect of cisplatin on ATR pathway activation was also distinct in luminal and basal-like breast cancer cells (Fig. S4b, c). In MDA-MB-231 cells, cisplatin exposure induced activation of ATR in both culture conditions, as opposed to MCF-7 cells, where a significant induction of Chk1 phosphorylation was detected only in 3D. Thus co-treatment with the ATR inhibitor (VE-821), in the MDA-MB-231 cell line, enhanced the cytotoxic effect of cisplatin in both 2D and 3D cellular contexts (Fig. S4d).

### REV3L mediates the cellular resistance to cisplatin and the effects of the ATR inhibitor in 3D-cultured breast cancer cells

**Fig. 6:**
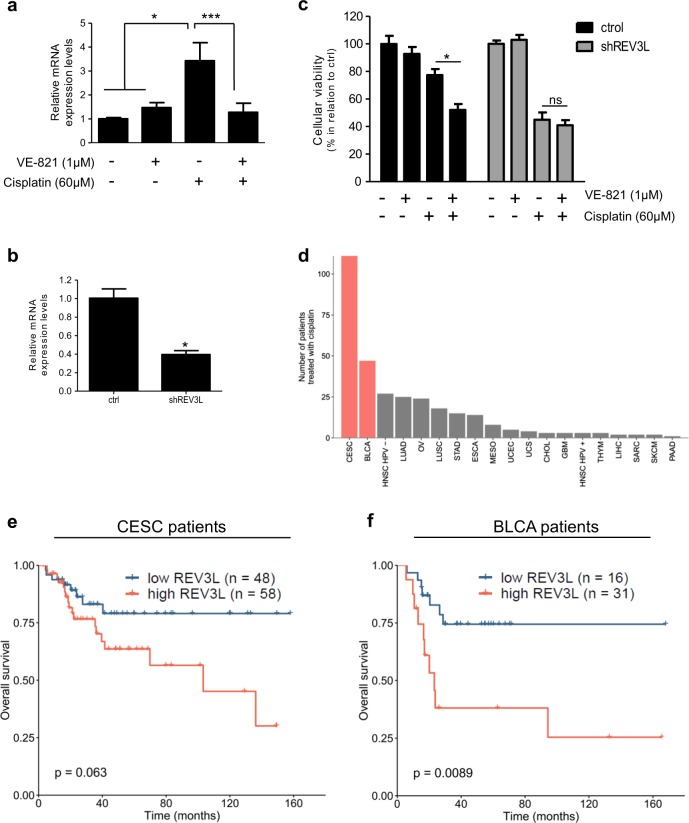
REV3L expression is important for cellular resistance to cisplatin in three-dimensional-cultured cells and cancer patients. **a**, **b** REV3L and GAPDH (endogenous control) mRNA expression levels assessment, by quantitative PCR assays, **a** in MCF-7 cells co-treated with VE-821 (ATR pharmacological inhibitor) and cisplatin for 24 h and **b** in REV3L-deficient (shREV3L) and scramble control (ctrl) MCF-7 cells. **c** Cellular viability of shREV3L and ctrl MCF-7 cells’ exposure to VE-821 and cisplatin for 72 h. **d**–**f** Analysis of Rev3L mRNA expression correlation with cancer patients’ response to cisplatin, using The Cancer Genome Atlas (TCGA) database. **d** Number of patients treated with cisplatin by cancer type. Cancer types with >40 cisplatin-treated patients were selected for analysis and are highlighted in red. **e**, **f** Kaplan–Meier survival curves of cisplatin-treated patients divided according to the mRNA expression of *REV3L* in pretreatment biopsies of **e** cervical squamous cell carcinoma and endocervical adenocarcinoma (CESC) and **f** bladder urothelial carcinoma (BLCA). **a**–**c** The results are presented as mean ± SEM from two independent experiments performed in triplicate. **a** One-way analysis of variance (ANOVA) followed by Tukey post-test, **b** Student *t* test, and **c** two-way ANOVA and Bonferroni post-hoc test were used for statistical analysis and the differences were considered significant for *P ≤ 0.05, **P ≤ 0.01 and ***P ≤ 0.001. ns highlights non-significant changes. **e**, **f** Comparisons were performed using log-rank test. **d** Types of cancer presented: CESC, BLCA, head and neck cell carcinoma HPV− (HNSC HPV−), lung adenocarcinoma (LUAD), ovarian serous cystadenocarcinoma (OV), lung squamous cell carcinoma (LUSC), stomach adenocarcinoma (STAD), esophageal carcinoma (ESCA), mesothelioma (MESO), uterine corpus endometrial carcinoma (UCEC), uterine carcinosarcoma (UCS), cholangiocarcinoma (CHOL), glioblastoma multiforme (GBM), head and neck cell carcinoma HPV+ (HNSC HPV+), thymoma (THYM), liver hepatocellular carcinoma (LIHC), sarcoma (SARC), skin cutaneous melanoma (SKCM), pancreatic adenocarcinoma (PAAD)

Given that cisplatin treatment led to transcriptional upregulation of TLS DNA polymerases (Fig. 4b–d) in 3D culture context, we checked whether ATR inhibition could also block this 3D-mediated effect. The mRNA expression levels of Pol eta, REV3L, and Pol iota (TLS DNA polymerases whose mRNA levels were modulated by cisplatin; Fig. 4b–d) were assessed in 3D-cultured MCF-7 cells treated with VE-821 and cisplatin. Co-treatment with VE-821 significantly reverted the cisplatin-associated increase of REV3L transcriptional levels (Fig. 6a). Non-significant effects of VE-821 on Pol eta and Pol iota mRNA levels were observed (data not shown).

To determinate the REV3L role in cellular survival to cisplatin and on VE-821 sensitization effect, we generated MCF-7 cells deficient for REV3L expression (shREV3L). Transduction with lentivirus carrying a specific shRNA sequence led to significant decrease of the REV3L mRNA expression levels, in relation to scramble control cells (ctrl) (Fig. 6b). Cellular viability assays demonstrated that 3D-cultured shREV3L cells are more sensitive to cisplatin treatment than the crtl ones (Fig. 6c). Furthermore, co-treatment with VE-821 did not induce any increment on cellular sensitivity to cisplatin of the shREV3L cells, suggesting that REV3L is required for the ATR inhibitor effect on cisplatin cellular viability.

Next, by using of publicly available databases, we evaluated whether the Rev3L mRNA expression levels could predict the cancer patients’ response to cisplatin treatment. We used data from patients diagnosed with cervical squamous cell carcinoma and endocervical adenocarcinoma (CESC) and bladder urothelial carcinoma (BLCA); since these were the only cancer types with data for cisplatin treatment available for >40 patients (Fig. 6d). For the Kaplan–Meier survival curves, CESC and BLCA cisplatin-treated patients were categorized according to the mRNA expression levels of Rev3L detected in pre-treatment biopsies (Fig. 6e, f, respectively). The data suggest that high expression of Rev3L is correlated with decreased overall survival, corroborating our findings that Rev3L is central for cisplatin resistance.

## Discussion

Similar to patients, the present study shows that 3D structures formed by breast cancer cells grown in reconstituted basement membrane are highly resistant to cisplatin’s cytotoxic effects^7,8^. However, when cultured on a flat surface, the same cells became highly sensitive to cisplatin. Accordingly, previous results from our group have also shown that chemoresistance is a 3D microenvironment-dependent process^4^. Under 2D culture conditions, breast cancer cells are resistant to doxorubicin, an anticancer drug that, differently from cisplatin, is part of breast cancer treatment regimens^4,7^. In turn, when exposed to doxorubicin in a 3D context, these cells acquire the expected high sensitivity to this drug, as seen in patients^4^. Therefore, although cellular growth in 3D-rBM can induce opposite effects depending of the anticancer agent tested, the tumor cells response in the 3D context seems to better reflect the outcome in patients.

Unexpectedly, our results revealed that 3D-cultured breast cancer cells present impaired cisplatin–DNA adduct repair. Nucleotide excision repair (NER) is the main DNA repair pathway responsible for removing cisplatin-induced DNA damage^24^. Depending on how the lesion is recognized, NER is subdivided into two mechanisms: global genome repair (GGR) and transcription-coupled repair (TCR)^25^. Owing to TCR-NER, repair of cisplatin damage is much higher for transcriptionally active DNA regions^26^. However, it has already been shown that cells cultured in the presence of a laminin-rich ECM-rBM undergo extensive chromatin histone deacetylation, chromatin condensation, and global gene expression reduction^27,28^. Therefore, changes on chromatin organization might underlie the persistence of the cisplatin-induced DNA damage, detected in 3D cultures.

We demonstrated that breast cancer cells were not killed, but became senescent when exposed to cisplatin in a 3D context. At first, senescence was considered a tumor-suppressive process, since it restricts proliferation of damaged cells^29^. Moreover, in addition to preventing tumorigenesis, senescence induction is also an important cellular response to genotoxic chemotherapeutic agents^30^. However, much evidence suggest that senescent cells also display tumor-promoting activities^16^. Although they do not proliferate, senescent cells remain metabolically active and secrete a variety of proteins, which collectively are known as senescence-associated secretory phenotype (SASP)^16,29^. SASPs can recruit immune cells and induce tumor cell removal, but, on the other hand, they can also promote cell proliferation and invasion of other tumor cells through a paracrine signaling, creating a pro-tumoral niche^31^. We detected, indeed, a substantial increase of IL-6 expression in 3D-cultured MCF-7 cells upon long periods of treatment with cisplatin. This SASP is a pro-inflammatory cytokine associated with DNA damage-induced senescence in several models, including epithelial cells^17^. Also, it is suggested that IL-6 is a key mediator of the SASP’s pro-tumor phenotype^32^.

**Fig. 7:**
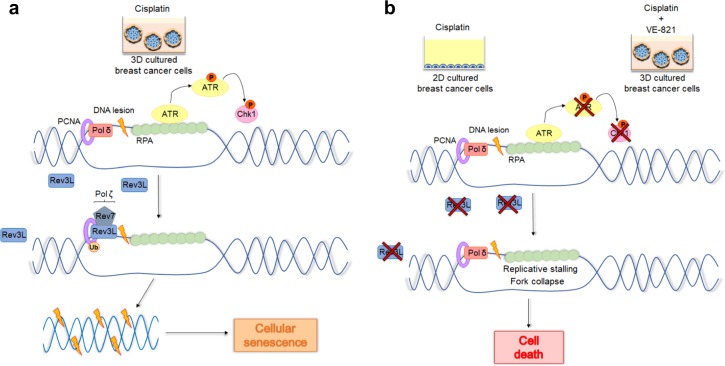
Proposed molecular mechanism of three-dimensional (3D) microenvironment-dependent breast cancer cell response to cisplatin. **a** Cells treated with cisplatin in a 3D context present significant induction of ATR-Chk1 pathway, which in turn is essential for REV3L upregulation, translesion DNA synthesis (TLS), and cellular survival. In contrast, **b** under two-dimensional-cultured conditions, no significant levels of p-Chk1 and REV3L induction were detected, leading to replication fork obstruction and elevated cellular sensitivity to this chemotherapy. Through a similar mechanism, blockage of cisplatin-mediated induction of ATR-Chk1 signaling, by co-treatment with VE-821, reverts the 3D-microenvironmet-associated resistance to cisplatin

Here we found that the amount of DNA damage generated by cisplatin is not different in 2D- and 3D-cultured cells. However, the DNA lesion tolerance capacity is very distinct between these culture conditions. The 3D culture-mediated resistance to cisplatin is highly correlated with TLS activity (Fig. 7a). On the other hand, MCF-7 cells exposed to cisplatin in a flat plastic surface seem to lose its enhanced capacity of replicate cisplatin–DNA adducts (Fig. 7b). Therefore, this study shows, for the first time, that the cell–ECM interaction can affect replication fork progression and, consequently, replicative stress. Our data corroborate the model of “dynamic reciprocity” between ECM and nucleus, which postulates a dynamic and bidirectional mechanism of interaction among the ECM, cytoskeleton, nuclear matrix, and chromatin^33^.

In addition, we also demonstrate a cisplatin-mediated induction of REV3L expression dependent of ATR. The relevance of REV3L in decreasing cellular responsiveness to cisplatin is already well established^34–36^. In this sense, increased levels of cisplatin-induced cell death were achieved by depletion of REV3L expression in several cancer models, including glioma, lung, and cervical cells^34–36^. Here it was demonstrated that REV3L downmodulation displays a synergic effect when combined with cisplatin also in breast cancer model.

In our proposed mechanism, ATR-Chk1 signaling plays a central role on cellular resistance to cisplatin (Fig. 7). In fact, it is already well established that cancer cells can increase resistance to the lethal effects of genotoxic anticancer agents by activating DDR signaling^10^. However, this is the first time that the 3D structure impact on DDR stimulation is demonstrated. Here we show that activation of ATR-Chk1 pathway in response to cisplatin treatment is facilitated by cell growth in 3D culture (Fig. 7a). Consequently, 3D-cultured breast cancer cells presented low sensitivity to this drug. In turn, increased chemotherapy efficacy was achieved by impairment of cisplatin-mediated induction of Chk1 phosphorylation (best-characterized ATR effector) through cell growth in monolayer or co-treatment with an ATR pharmacological inhibitor (Fig. 7b)^19^. Because of the high frequency of tumor resistance and side effect induction in response to cisplatin, its combination with other drugs have been applied as potential therapeutic strategies for many types of human cancer^37^. Several studies obtained promising results regarding the use of ATR inhibitors for decreasing cisplatin resistance^38,39^. Thus clinical trials for testing of potent ATR inhibitors in combination with cisplatin or other chemotherapies have been performed^40^. Our results suggest that ATR blockage might be a latent strategy to enable the cisplatin use also for breast cancer patients. This conclusion was only possible using 3D cell cultures, since under monolayer conditions neither non-significant ATR-Chk1 activation nor VE-821-sensitizing effect was detected in response to cisplatin. Therefore, the use of preclinical models, which include 3D-microenvironments that allow cell–cell and cell–ECM interactions akin to what cells experience in vivo in drug screening studies, can lead to more efficient development of therapeutic strategies for cancer patients.

## Material and methods

### Cell culture

The breast (MCF-7 and MDA-MB-231) and the lung (A549 and NCI-H23) cancer cells were maintained according the American Type Culture Collection (ATCC) recommendations. The 3D-rBM assays were performed as previously described^4^. For non-adherent 3D culture assay, poly-2-hydroxyethyl methacrylate (polyHEMA, Sigma-Aldrich, Saint Louis, MO, USA) was dissolved in 95% ethanol at 12 mg/ml. Plates were then coated at 0.8 mg/cm^2^ and air dried at 37 °C overnight. Also, 1 × 10^4^ cells/cm^2^ were seeded as single cells, on polyHEMA-coated plates, in Dulbecco’s modified Eagle’s medium supplemented with 2.5% fetal bovine serum. After 48 h (time for 3D structure formation), for the conditions in which the basement membrane effect was evaluated, the media was supplemented with 4% Matrigel^®^. The cellular viability in response to cisplatin (60 μM) was initiated 4 days after cell plating.

### Reagents

The cells were treated with cisplatin, chloroquine, AZ20, and/or VE-821 (Sigma-Aldrich). All thymidine analogs (bromodeoxyuridine (BrdU), CldU, and IdU) were purchased from Sigma-Aldrich. For immunoblot analysis, we used the following antibodies: p53 (Agilent Technologies, Santa Clara, CA, USA), GAPDH (Santa Cruz Biotechnology, Dallas, TX, USA), LC3B (Cell Signaling Technology, Danvers, MA, USA), phospho-Chk1 (Ser345) (Santa Cruz Biotechnology), and Chk1 (Cell Signaling Technology). For specific detection of cisplatin–DNA adducts by slot-blot assays, the antibody for cisplatin-modified DNA (Abcam, Cambridge, UK) was used. Antibodies against γ-H2AX (Merck Millipore, Burlington, MA, USA) and BrdU (Agilent Technologies) were used in flow cytometric analyses.

### Cellular viability and death assays

Cellular viability analyses were performed using the Cell Proliferation Kit II (XTT) (Roche, Basel, Switzerland) according to the manufacturer’s instructions. Detection of cell death by fluorescence microscopy was performed as previously described by Moraes and collaborators^41^. This cell death assay is based on the different profile of staining for fluorescein di-acetate (FDA), propidium iodide (PI), and Hoechst 33342 (HO) by viable, apoptotic, and necrotic cells.

### DNA fiber assay

The progression of replication forks after cisplatin and/or VE-821 (ATR inhibitor) treatment was evaluated by DNA fiber assays; for this, MCF-7 cells were exposed to a 20-min pulse of 20 µM CldU, followed by co-incubation (60 min) with 200 µM IdU and cisplatin (150 and 300 μM) and/or VE-821 (1 µM). After these incubations, the cells were harvested on ice by mechanical scraping and lysed (0.5% SDS, 200 mM Tris-HCl pH 7.4, and 50 mM EDTA) on a glass slide. The slides were then tilted to allow DNA to spread along the slide. The samples were fixed in methanol–acetic acid (3:1) and washed with 70% ethanol. DNA was incubated with methanol for 5 min, washed with phosphate-buffered saline (PBS), denatured with 2.5 M HCl, and blocked with 5% bovine serum albumin (BSA; Sigma-Aldrich). IdU and CldU were stained at the same time using mouse anti-BrdU antibody (BD Biosciences, Franklin Lakes, NJ, USA) and rat anti-BrdU antibody (Bio-Rad Laboratories, Hercules, CA, USA), respectively, in PBS-T-BSA (0.05% Tween-20, 1% BSA). The slides were then washed with PBS-T followed by anti-mouse Alexa Fluor 594 and anti-rat Alexa Fluor 488 secondary antibodies (Thermo Fisher Scientific, Waltham, MA, USA). The slides were mounted using Fluoroshield (Sigma-Aldrich), and DNA fibers were imaged by a fluorescent microscope (Axiovert 200, Zeiss, Oberkochen, Germany) at a magnification of ×1000. Analyses were performed using the Zeiss LSM Browser Software. All experiments were performed at least twice, and a minimum of 150 fibers was counted for each slide.

### Flow cytometric assays for cell cycle profile and DNA damage analysis

Flow cytometric assays were performed to assess BrdU (Sigma-Aldrich) incorporation and PI staining according to previously described method^42^. For immunodetection of γ-H2AX, a well-established DNA damage marker, flow cytometry was also performed as previously described by Quinet and collaborators^42^.

### β-Galactosidase assay

For detection of the β-galactosidase activity at pH 6, recognized methodology for cellular senescence measurement, the Senescence β-Galactosidase Staining Kit (Cell Signaling Technology) was used according to the manufacturer’s instruction. Using the Image J^®^ processing program, the relative intensity of the β-galactosidase staining was quantified from images obtained (×20 objective lens) in EVOS Cell Imaging Systems (Thermo Fisher Scientific).

### Quantitative PCR analysis

PureLink^TM^ RNA Mini Kit (Thermo Fisher Scientific) was used for total RNA extraction, whose quality and quantity were evaluated using the NanoDrop^TM^ 1000 Spectrophotometer (Thermo Fisher Scientific). Each RNA sample was treated with DNase (Promega, Madison, WI, USA) and further used as template in reverse transcription reactions (using the High-Capacity cDNA Reverse Transcription Kit, Thermo Fisher Scientific) for cDNA synthesis. Quantitative PCR reactions were performed using Power SYBR Green PCR Master Mix and the 7500 Real-Time PCR Systems, both purchased from Thermo Fisher Scientific. The following primer (Exxtend, Campinas, SP, Brazil) sequences were used in the quantitative PCR assays: p21 (forward: TGGAGACTCTCAGGGTCGAAA and reverse: CGGCGTTTGGAGTGGTAGAA, IL-6 (forward: GGCACTGGCAGAAAACAACC and reverse: GCAAGTCTCCTCATTGAATCC), Pol eta (forward: GGTCAGTCCCACAGCTCTTC and reverse: GACAGAGGCCCCTAGCTTTC), REV3L (forward: TTTGTGCCAGCAACAGAAAG and reverse: CTGGGATCCATCGCTGTAGT), Pol iota (forward: GTCGTGAGAGTCGTCAGTGC and reverse: GCTTGCCAGAGCGTGAAGTA), REV1 (forward: CCCAGACATCAGAGCTGTATAAT and reverse: CTTCCTGTGCCTCTGTTACTT), Pol kappa (forward: AGCCATGCCAGGATTTATTG and reverse: GGATCGTTCATGCTCACTCA), and GAPDH (forward: ACCCACTCCTCCACCTTTGA and reverse: CTGTTGCTGTAGCCAAATTCGT). Relative mRNA expression levels were calculated according to the 2^−∆∆Ct^ methodology^43^.

### The Cancer Genome Atlas (TCGA) data analyses

TCGA RNA-seq and clinical data were downloaded from the UCSC XENA Browser in February 2019. Gene expression data were generated using the Illumina HiSeq 2000 RNA sequencing platform, quantified as HT-Seq FPKM-UQ, and log_2_(FPKM-UQ + 1) transformed^44^. In the survival analysis, gene expression cutoffs used for sample dichotomization were selected using a log-rank test-based strategy that identifies the most significant split^45,46^.

### Statistical analysis

Statistical analysis was performed using the GraphPad Prism 5.0 software (GraphPad Software, Inc, La Jolla, CA, USA). In the graphs, the results are presented as mean ± SEM. Statistical significance was determined by Student *t* test, one-way analysis of variance (ANOVA) followed by Tukey post-test, or two-way ANOVA followed by Bonferroni post-test, depending of the number of conditions and groups to be compared. The experiments were repeated at least two times in triplicate.

## Supplementary information

Figure S1

Figure S2

Figure S3

Figure S4

Supplementary figure legends
