# Supplementary figures and images for: ATR mediates cisplatin resistance in 3D-cultured breast cancer cells via translesion DNA synthesis modulation
Source: Cell Death Dis. 2019 Jun 12;10(6):459. doi: 10.1038/s41419-019-1689-8 (PMC6561919; doi:10.1038/s41419-019-1689-8)

**a**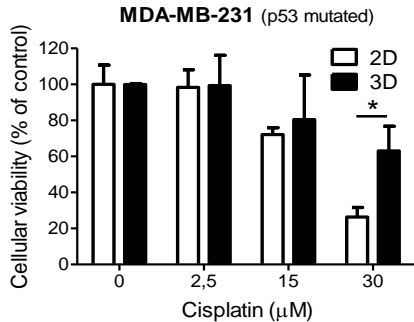**b**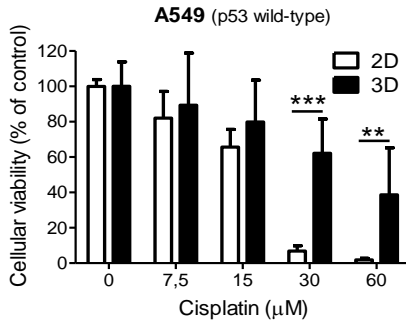**c**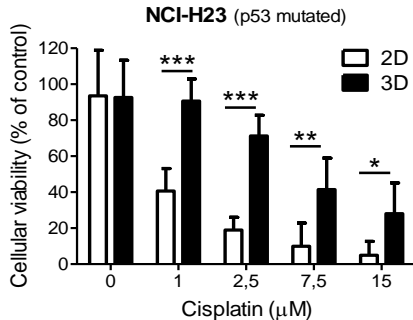

Supplement: Supplementary file 1 — Figure S1 [file 41419_2019_1689_MOESM1_ESM.pdf]

**a**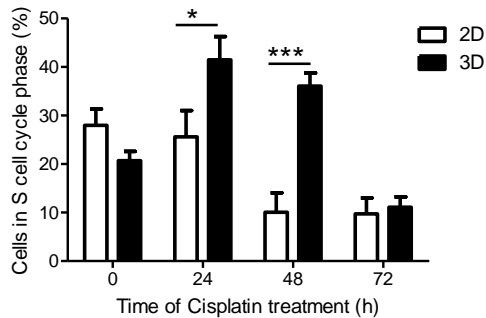**b**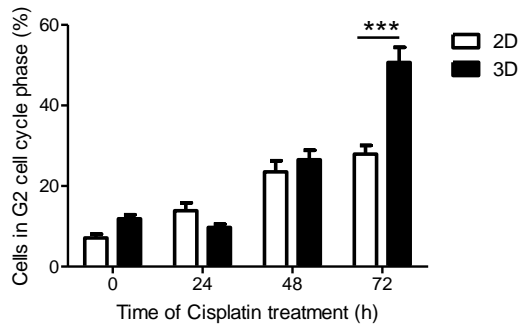**c**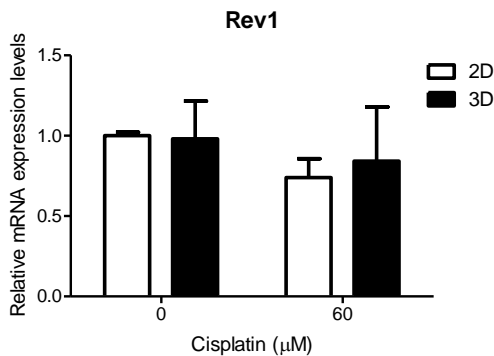**d**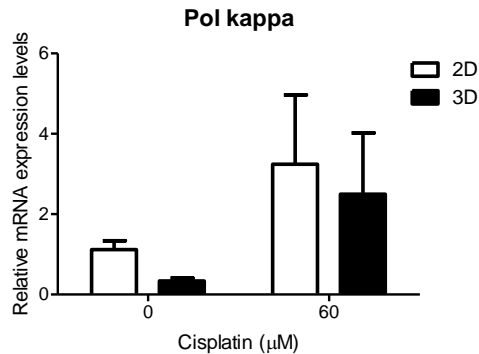

Supplement: Supplementary file 2 — Figure S2 [file 41419_2019_1689_MOESM2_ESM.pdf]

**a**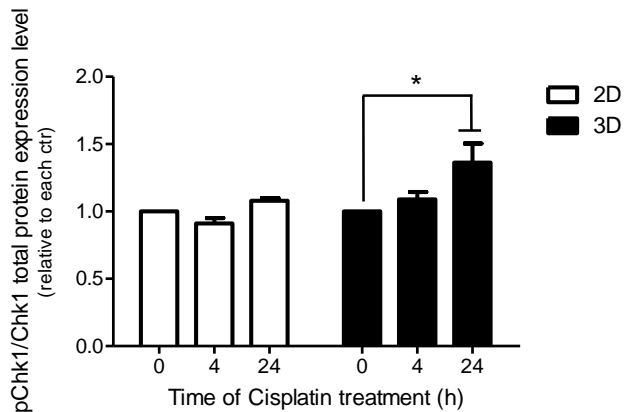**b**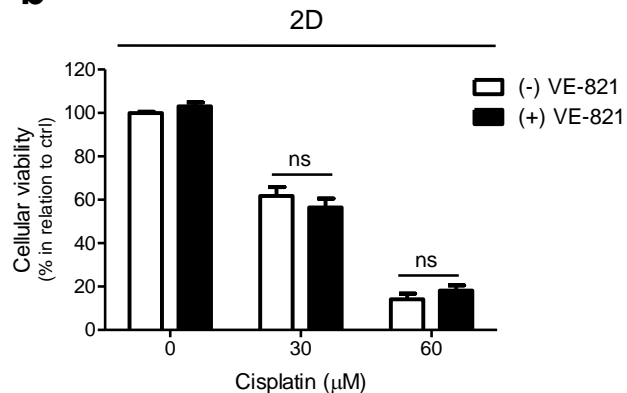**c**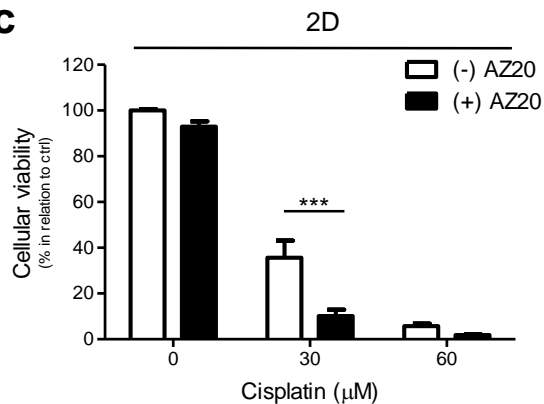**d**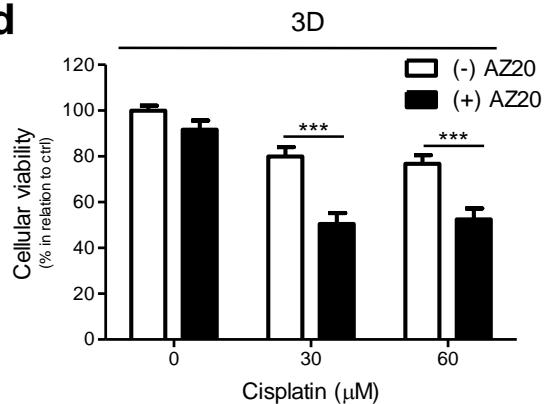

Supplement: Supplementary file 3 — Figure S3 [file 41419_2019_1689_MOESM3_ESM.pdf]

**a**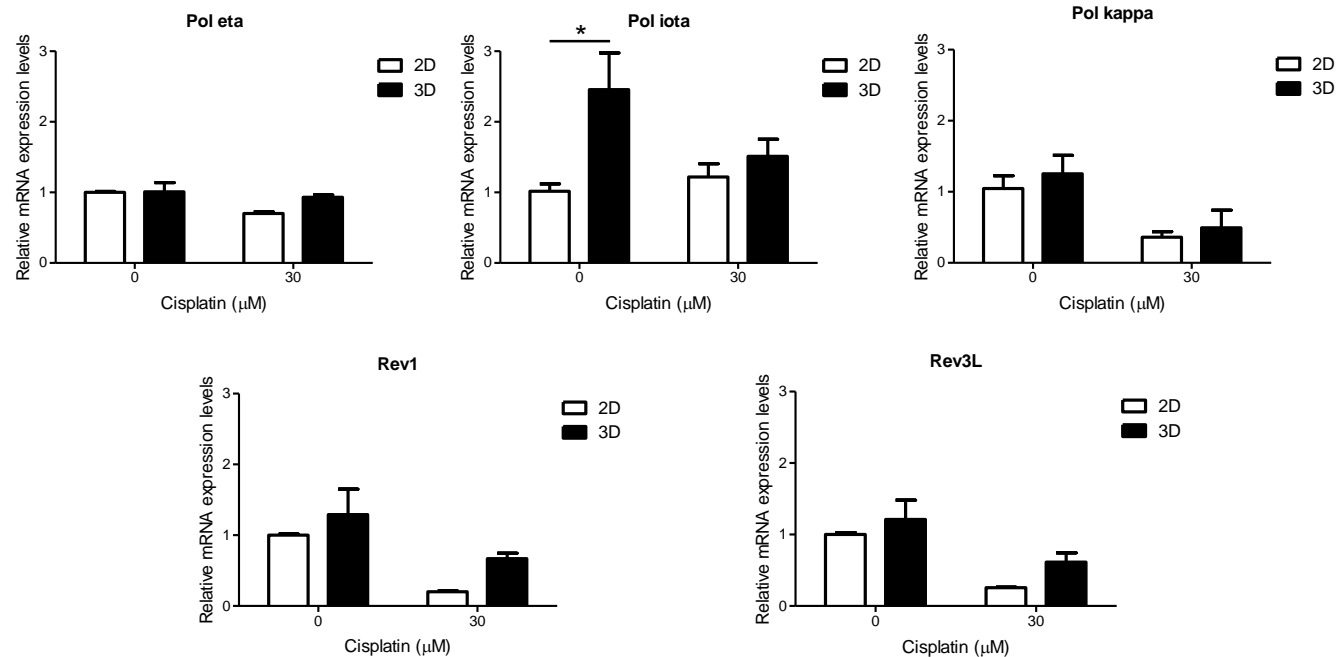**b**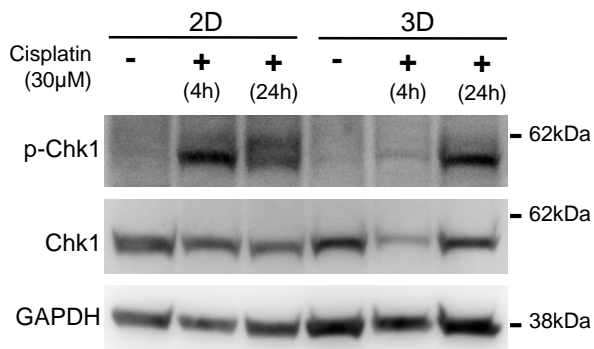**c**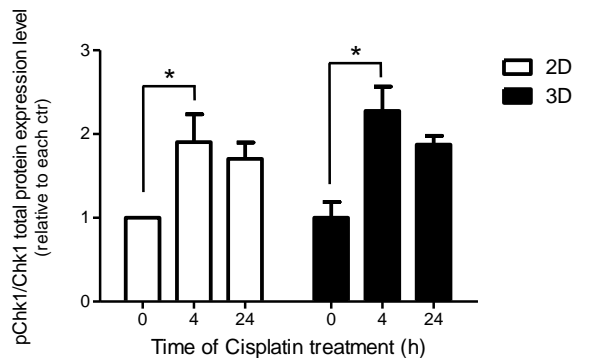**d**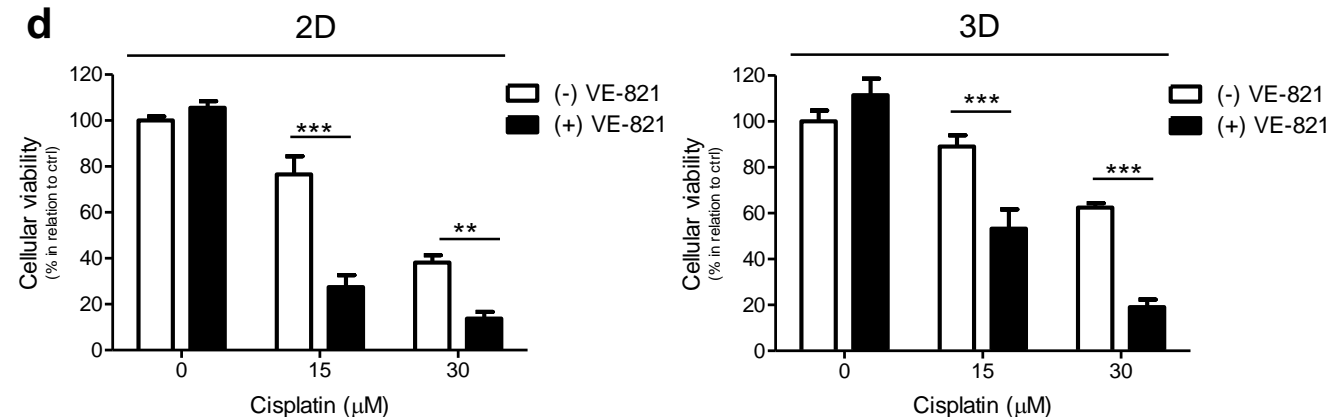

Supplement: Supplementary file 4 — Figure S4 [file 41419_2019_1689_MOESM4_ESM.pdf]
