# Supplementary material for: ATR mediates cisplatin resistance in 3D-cultured breast cancer cells via translesion DNA synthesis modulation
Source: Cell Death Dis. 2019 Jun 12;10(6):459. doi: 10.1038/s41419-019-1689-8 (PMC6561919; doi:10.1038/s41419-019-1689-8)
Supplement: Supplementary file 5 — Supplementary figure legends [file 41419_2019_1689_MOESM5_ESM.doc]

**Supplementary figures legends**

**Fig. S1** Independently of p53 status breast and lung cancer cells presented increased resistance to cisplatin when maintained in 3D culture. (**a-c**) Analysis of cellular viability to treatment with distinct doses of cisplatin (for 72 h) on (**a**) p53 mutated breast cancer cells (MDA-MB-231), (**b**) p53 wild-type (A549) and (**c**) p53 mutated (NCI-H23) lung cancer cells, through XTT methodology. In all graphs the results are presented as mean ± SEM from two independent experiments performed in triplicate. Two-way ANOVA and the Bonferroni post-hoc test were used for statistical analysis and the differences were considered significant for * *P* ≤ 0.05, ** *P* ≤ 0.01 and *** *P* ≤ 0.001.

**Fig. S2** Cell cycle progression and expression of translesion DNA polymerases in MCF-7 cells treated with cisplatin under 2D and 3D cell culture conditions. (**a** and **b**) Quantification of percentage of cells in (**a**) S and (**b**) G2 cell cycle phases upon distinct times of treatment with 60 μM of cisplatin, using double staining with BrdU and PI followed by flow cytometry. (**c** and **d**) qPCR assays for detection of mRNA expression levels of (**c**) REV1 and (**d**) Pol kappa upon treatment with cisplatin (24 h). GAPDH mRNA expression was used as endogenous control for qPCR assays. In all graphs the results are presented as mean ± SEM from at least two independent experiments performed in duplicate. Two-way ANOVA and the Bonferroni post-hoc test were used for statistical analysis and the differences were considered significant for * *P* ≤ 0.05, ** *P* ≤ 0.01 and *** *P* ≤ 0.001.

**Fig. S3** Quantification of p-Chk1/Chk1 ration in 2D and 3D-cultured MCF-7 cells treated with cisplatin and effect of ATR inhibition on viability of cells exposure to cisplatin under distinct culture conditions. (**a**) Densitometric analysis of p-Chk1 (S345) protein levels normalized to the Chk1 total. Each cisplatin-treated condition was normalized regarding its respective untreated control (2D or 3D). (**b**) Cellular viability analysis of 2D-cultured cells treated with 1 μM of VE-821 (ATR pharmacological inhibitor) and different doses of cisplatin for 72 h, by XTT assays. (**c** and **d**) Effect of co-treatment with 500 nM of AZ20 (another ATR pharmacological inhibitor) on cellular viability of MCF-7 cells exposed to cisplatin (30 and 60 μM) under (**c**) 2D and (**d**) 3D culture conditions. In all graphs the results are presented as mean ± SEM from at least two independent experiments. (**a**) One-way ANOVA followed by Tukey post-test and (**b**, **c** and **d**) Two-way ANOVA and the Bonferroni post-hoc test were used for statistical analysis and the differences were considered significant for * *P* ≤ 0.05, ** *P* ≤ 0.01 and *** *P* ≤ 0.001. ns represents non-significant changes.

**Fig. S4** Effects of cisplatin in TLS DNA polymerases expression and ATR activation in MDA-MB-231 cells, cultured under in 2D and 3D cell culture contexts. (**a**) qPCR assays for detection of mRNA expression levels of Pol eta, Pol iota, Pol kappa, Rev1 and REV3L upon treatment with 30 μM of cisplatin (24 h). GAPDH mRNA expression was used as an endogenous control for qPCR assays. (**b**) Immunoblots for p-Chk1 (Ser345), total Chk1 and GAPDH (loading control) in protein lysates from cells treated with 30 μM cisplatin for 4 and 24 h, under 2D and 3D culture conditions. (**c**) Densitometric analysis of p-Chk1 (S345) protein levels normalized to the Chk1 total. Each cisplatin-treated condition was normalized regarding its respective untreated control (2D or 3D). (**d**) Cellular viability analysis of 2D and 3D-cultured MDA-MB-231 cells, co-treated with 1 μM of VE-821 (ATR pharmacological inhibitor) and cisplatin (15 and 30 μM) for 72 h, by XTT assays. In all graphs the results are presented as mean ± SEM from at least two independent experiments. (**c**) One-way ANOVA followed by Tukey post-test and (**a** and **d**) Two-way ANOVA and the Bonferroni post-hoc test were used for statistical analysis and the differences were considered significant for * *P* ≤ 0.05, ** *P* ≤ 0.01 and *** *P* ≤ 0.001.
